# OP84 How Environmentally Sensitive Are Health Technology Assessment Value Frameworks? A Scoping Review

**DOI:** 10.1017/S0266462325101384

**Published:** 2025-12-29

**Authors:** Veronica Alfie, Fernando Argento, Carla Colaci, Andrea Alcaraz, Andrés Pichon-Riviere, Federico Augustovski

## Abstract

**Introduction:**

Climate change is a global concern. Medical technology, from production to disposal, has diverse environmental impacts. Many healthcare systems use value frameworks to inform transparent decision-making. Our objective was to review how healthcare systems currently consider environmental sustainability in health technology assessment (HTA) value frameworks.

**Methods:**

A scoping review was conducted to identify value frameworks focused on the assessment of any type of medical technology from 2000 to 2024. We examined main literature databases, HTA agency websites, and gray literature, with no language restrictions. Two researchers extracted dimensions from the surveyed frameworks. A wide range of value frameworks were included, and we surveyed whether environmental impact (broad term) was included within the dimensions of value. Value framework characteristics extracted included variables like origin, intended use, and the presence of evaluation or explicit weighting methods for a technology’s environmental impact.

**Results:**

Forty-eight value frameworks were identified. Sixteen percent (n=8) mentioned the environmental impact of the technology. Three were developed by HTA agencies (Australia, Canada, and the UK) and the rest were from other stakeholders in Europe, Latin America, the USA, and a global alliance. Except for two frameworks focused on diagnostic technologies, most were geared toward healthcare technologies more broadly. Only one framework suggested an assessment tool for environmental impact. Four frameworks provided a conceptual definition of this dimension without tools or metrics for assessing it, while the remaining three solely listed this dimension.

**Conclusions:**

Despite the increasing global relevance of the effect that human activities and natural events have on the environment, only a minority of value frameworks considered the incorporation of environmental impact of the healthcare technologies they assess. This study’s key contribution is surveying current consideration of environmental impact in health systems, providing a starting point for future actions.

